# Optical Biopsy and Diagnosis of Gastric Mucosa-Associated Lymphoid Tissue-Type Lymphoma by Probe-Based Confocal Laser Endomicroscopy

**DOI:** 10.3390/diagnostics16101451

**Published:** 2026-05-10

**Authors:** Mengmeng Zhang, Xinxin Mao, Xi Wu, Wen Shi, Yunlu Feng, Aiming Yang

**Affiliations:** 1 Department of Gastroenterology, Peking Union Medical College Hospital, Chinese Academy of Medical Sciences and Peking Union Medical College, Beijing 100730, China; 2 Department of Pathology, Peking Union Medical College Hospital, Chinese Academy of Medical Sciences and Peking Union Medical College, Beijing 100730, China

**Keywords:** gastric mucosa-associated lymphoid tissue, magnifying endoscopy, probe-based confocal laser endomicroscopy

## Abstract

The endoscopic findings of gastric mucosa-associated lymphoid tissue (MALT) lymphoma are highly nonspecific and the sampling error or false-negative probabilities during conventional biopsy make its diagnosis more challenging. Confocal laser endomicroscopy is a novel technology which allows in vivo microscopic analysis of gastrointestinal mucosa. Here we present a case of gastric MALT lymphoma by targeted biopsy guided by magnifying endoscopy and probe-based confocal laser endomicroscopy.

**Figure 1:**
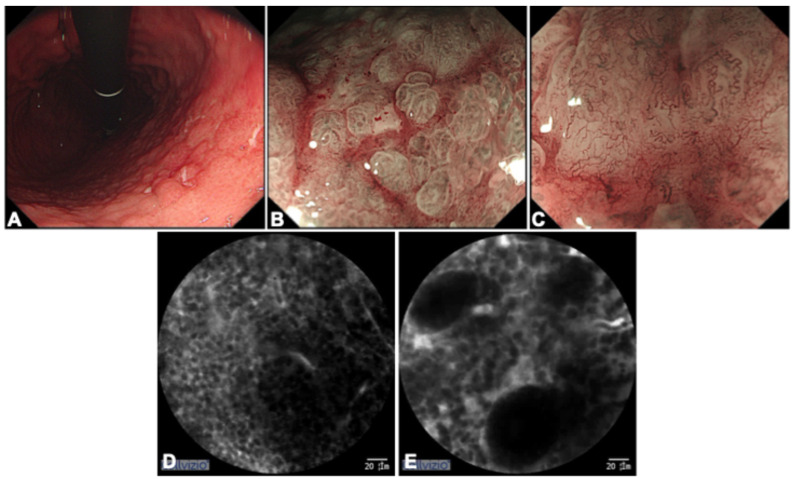
A 55-year-old female with suspected early gastric carcinoma during conventional gastroscopy was admitted without clinical symptoms. We proceeded with the observation with white light endoscopy (WLE) and magnifying endoscopy with narrow-band imaging (ME-NBI) with a high-resolution gastroscope (GIF-Q260, Olympus, Tokyo, Japan). Panel (**A**): WLE revealed that with the gastritis-like background mucosa, there were nodular elevations and shallow ulcers without clear boundaries in the lesser curvature of the gastric body, where the microsurface pattern (MSP) is absent and the microvascular pattern (MVP) appears tortuous and thickened in a tree-like pattern under ME-NBI in Panels (**B**,**C**). Subsequently, fluorescein-aided probe-based confocal laser endomicroscopy (pCLE) was performed with the GastroFlex UHD miniprobe (Cellvizio; Mauna Kea Technologies, Paris, France). Within 30 s to 8 min after intravenous fluorescein administration, we scanned the area within 2 cm around the mucosal lesion in the lesser curvature of the gastric body using the confocal probe. The probe was first used on the relatively normal mucosa around the lesion and then was moved to suspicious area. The examination lasted approximately 5 min. One pCLE video sequence was obtained and recorded. Panels (**D**,**E**): pCLE revealed the destruction of normal mucosal structure, replaced by dark areas where small roundish cells appear in a dense arrangement and infiltrate the lamina propria invading the epithelial layer in the fundus and lesser-curvature gastric body area (the pCLE video is provided in the Supplementary Materials). The *H. pylori* rapid urease test was negative and the ^13^C-urea breath test was positive.

**Figure 2:**
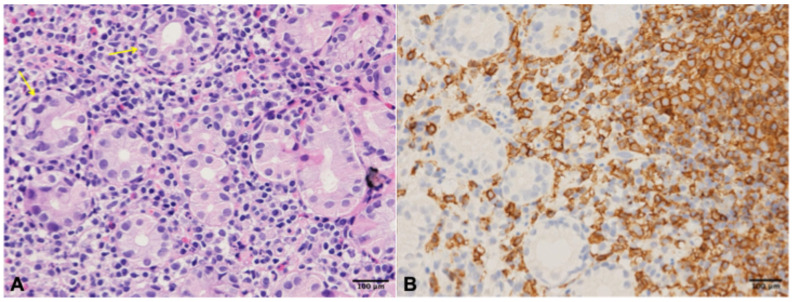
The characteristics of pCLE were in accordance with the targeted biopsy result. Panel (**A**): Hematoxylin and eosin staining showed multiple lymphoepithelial lesions in which lymphoma cells infiltrated the completely disrupted gastric epithelium (yellow arrows, original magnification ×200). Panel (**B**): The immunohistochemistry (original magnification ×200) revealed the lymphoma cells were positive for CD20 staining with a Ki-67 index of 20%, focally positive for Bcl-2, Bcl-6 and CD5, and negative for CD3, CD10, CD23, Cyclin D1 and SOX 11. Gastric mucosa-associated lymphoid tissue (MALT) lymphoma was finally diagnosed at Ann Arbor stage IE according to the patient’s PET/CT test, and she completed standardized *H. pylori* eradication therapy followed by negative ^13^C-urea breath test. As the mucosal lesion was prominent and the diagnosis was confirmed by biopsy, EUS was not performed in this patient. After 1 year, gastroscopy revealed significantly improved gastric mucosa with mildly irregular MVP and MSP (Supplementary Figure S1) and the pathology of the biopsy was mild nonspecific inflammation, evaluated as complete remission according to the GELA histological grading system. As the patient had already achieved remission, pCLE was not performed. The endoscopic findings of gastric MALT lymphoma are often nonspecific, including fold thickening, nodular appearances, erosions or ulcerations [1,2,3,4,5], especially in *H. pylori*-negative gastric MALT lymphoma, which has more small lesions without the undifferentiated carcinoma-like depressed lesions [6]. Confocal laser endomicroscopy is a novel technology which allows in vivo microscopic analysis of gastrointestinal mucosa [7]. Several case reports have suggested that pCLE can help recognize gastric MALT [8,9]. Dark cellular infiltrates have been reported in gastrointestinal carcinomas; however, carcinomas typically exhibit heterogeneous malignant cells with variable morphology instead of the cellular uniformity of small round cell infiltration [10]. As for the lymphoepithelial lesions and dense lymphoid infiltrates, these should be differentiated from lymphoid hyperplasia, chronic gastritis with prominent lymphoid follicles, or diffuse large B-cell lymphoma. Lymphoid hyperplasia and chronic gastritis with lymphoid follicles are benign conditions characterized by preserved glands and regular follicular structures [11]. In contrast, diffuse large B-cell lymphoma shows destruction of glandular structures with infiltration by large, pleomorphic malignant cells [3]. Through pCLE, targeted biopsy can improve the positive probabilities for MALT histological diagnosis. Previous studies have primarily relied on static images to identify potential features of MALT [10]. However, given that pCLE is used to observe mucosal microstructures in a dynamic manner, we provide the pCLE video in this case, enabling a more comprehensive and dynamic demonstration of its value in the diagnosis of gastric MALT. Additionally, follow-up endoscopic examination after *H. pylori* eradication therapy was performed in this case to further validate the diagnosis. As this patient responded well to *H. pylori* eradication therapy, genetic testing and follow-up pCLE were not performed, which is also one of the limitations of this case. Notably, pCLE typically requires the endoscopist to first identify the target lesion and then use pCLE to examine the microstructure, demanding greater clinical experience and technical skill. And due to the limited penetration depth of pCLE, there remain false-negative possibilities for MALT lesions located in deep layers. Furthermore, pCLE may prolong the examination time, so prior communication with the patient is necessary to ensure cooperation. In summary, pCLE, as an evolving technology, enables collection of real-time in vivo histological images of gastric mucosa, which might serve as a promising method to improve the accuracy of conventional biopsy but also requires further validation in identifying gastric MALT lymphoma.

## Data Availability

The data underlying this study are available from the corresponding author upon reasonable request.

## Supplementary Materials

The following supporting information can be downloaded at https://www.mdpi.com/article/10.3390/diagnostics16101451/s1. Figure S1: White light endoscopy and magnifying endoscopy with narrow-band imaging after *H. pylori* eradication therapy. (**A**) White light endoscopy presents improvement of the previously identified nodular elevated lesion in the lesser curvature of the gastric body. (**B**,**C**) Magnifying endoscopy combined with narrow-band imaging shows the mildly irregular microsurface and microvascular structure. Video S1: Probe-based confocal laser endomicroscopy in the patient with suspected early gastric carcinoma. The fluorescein sodium permeated into the interstitial space and showed the highlighted space among the glands with increased distance between glands, indicating active gastritis in the background mucosa. A mass of small roundish cells with similar size and morphology infiltrate in a dense arrangement in the gastric fundus and lesser curvature of the gastric body. In the lesser-curvature gastric body area, dark small infiltrates are identified in the completely disrupted gastric epithelium, namely the typical “lymphoepithelial infiltration” of MALT lymphoma.
