# Supplementary figures and images for: Optical Biopsy and Diagnosis of Gastric Mucosa-Associated Lymphoid Tissue-Type Lymphoma by Probe-Based Confocal Laser Endomicroscopy
Source: Diagnostics (Basel). 2026 May 10;16(10):1451. doi: 10.3390/diagnostics16101451 (PMC13206505; doi:10.3390/diagnostics16101451)

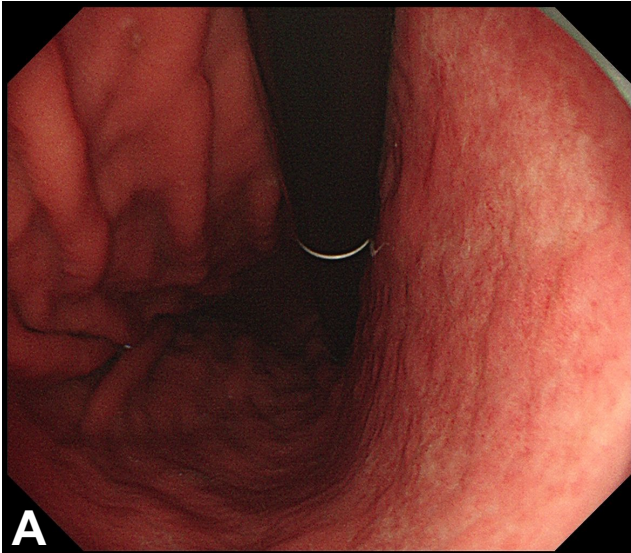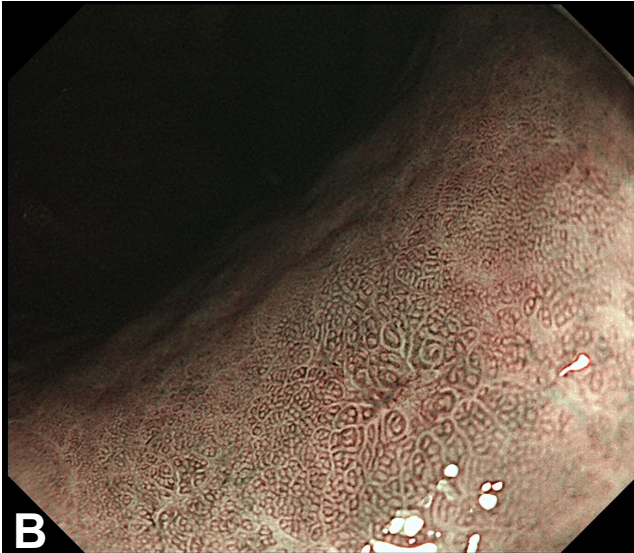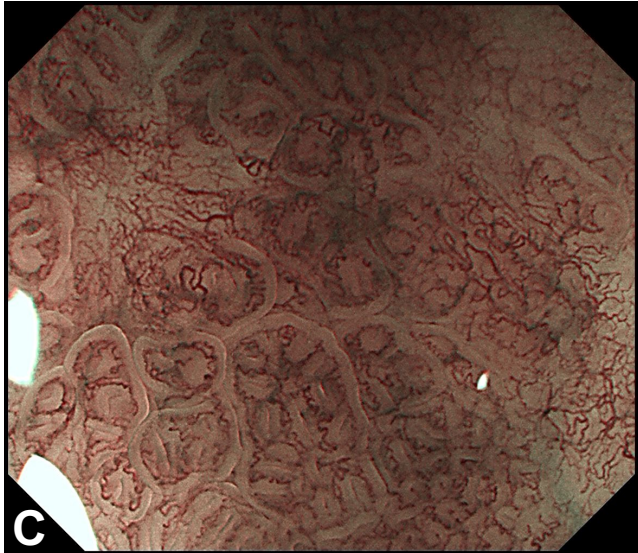

Supplement: Supplementary file 1 [file diagnostics-16-01451-s001.zip › Supplementary Figure S1.pdf]
